# Seaweeds as Valuable Sources of Essential Fatty Acids for Human Nutrition

**DOI:** 10.3390/ijerph18094968

**Published:** 2021-05-07

**Authors:** Carolina P. Rocha, Diana Pacheco, João Cotas, João C. Marques, Leonel Pereira, Ana M. M. Gonçalves

**Affiliations:** 1 University of Coimbra, MARE—Marine and Environmental Sciences Centre, Department of Life Sciences, Calçada Martim de Freitas, 3000-456 Coimbra, Portugal; carolina.vpr@gmail.com (C.P.R.); diana.pacheco@uc.pt (D.P.); jcotas@uc.pt (J.C.); jcmimar@ci.uc.pt (J.C.M.); leonel.pereira@uc.pt (L.P.); 2 Department of Biology and CESAM, University of Aveiro, 3810-193 Aveiro, Portugal

**Keywords:** indigenous and non-indigenous seaweeds, marine resources, fatty acids, nutritional value, human health promoter

## Abstract

The overexploitation of terrestrial habitats, combined with the ever-growing demand for food, has led to the search for alternative food sources. The importance of seaweeds as food sources has been growing, and their potential as sources of fatty acids (FA) make seaweeds an interesting feedstock for the food and nutraceutical industries. The aim of this study is to assess the potential of five red seaweeds (*Asparagospis armata*, *Calliblepharis jubata*, *Chondracanthus teedei* var. *lusitanicus*, *Gracilaria gracilis*, and *Grateloupia turuturu*) and three brown seaweeds (*Colpomenia peregrina*, *Sargassum muticum* and *Undaria pinnatifida*), harvested in central Portugal, as effective sources of essential FA for food or as dietary supplements. FA were extracted from the biomass, transmethylated to methyl esters, and analyzed through gas chromatography-mass spectrometry. *G. gracilis* presented the highest content of saturated fatty acids (SFA) (41.49 mg·g^−1^), whereas *C. jubata* exhibited the highest content of highly unsaturated fatty acids (HUFA) (28.56 mg·g^−1^); the three *G. turuturu* life cycle stages presented prominent SFA and HUFA contents. Omega-6/omega-3 ratios were assessed and, in combination with PUFA+HUFA/SFA ratios, it is suggested that *C. jubata* and *U. pinnatifida* may be the algae with highest nutraceutical potential, promoting health benefits and contributing to a balanced dietary intake of fatty acids.

## 1. Introduction

Lipids are essential nutrients for human health. However, it is necessary to ensure a balanced intake in appropriate quantities and in combination with other important nutrients, such as vitamins, carbohydrates, proteins, and minerals [1,2]. Lipids’ primary role is to provide energy, but they are also needed for the maintenance of cell membrane integrity and hormone production [3]. Moreover, lipids are essential to transport and absorb fat-soluble vitamins (i.e., A, D, E, and K).

Diseases such as obesity, diabetes, dyslipidemia, and cardiovascular-related co-morbidities have all been linked to a high saturated lipid intake, mostly saturated fatty acids (SFA) [4,5]. Nowadays, fast food is one of the most consumed food types in the world, and contains a high amount of saturated fats. As a consequence, harmful effects on human health are observed related to this type of diet [6,7].

There is a growing need to evaluate novel food sources that do not imply the overexploitation of terrestrial ecosystems [8] to release some pressure over these systems. Therefore, seaweeds present an innovative feedstock for the food market as a fatty acid (FA) source. Despite the low total lipid concentration that seaweeds contain, they have a significant amount of essential unsaturated fatty acids (UFA), which are pivotal for human welfare [6,9]. Particularly, seaweeds synthesize omega-3 and -6 (ω-3 and ω-6, respectively), polyunsaturated fatty acids (PUFA), and highly unsaturated fatty acids (HUFA), as well as monounsaturated fatty acids (MUFA) [10], which have already been demonstrated to play a key role in human metabolism (essential fatty acids—EFA), being involved in cell growth and metabolic pathways, contrarily to SFA, which serve mainly as energy sources [11].

The lipidic profile of seaweeds differs between species [6], and thus, there is a need to evaluate different seaweed species to understand their potential for industrial application and exploitation, such as food products or as dietary supplements [9,12].

The overgrowth of non-indigenous seaweed species is currently endangering aquatic systems, threatening coastal fauna, flora, and the ecosystem services provided [13,14]. Several non-indigenous macroalgae have been reported as serious threats to the marine environment on the Iberian Peninsula, including the red seaweeds *Asparagopsis armata* and *Grateloupia turuturu*, as well as the brown seaweeds *Sargassum muticum*, *Undaria pinnatifida*, and *Colpomenia peregrina* [15]. Therefore, not only native species, but also non-indigenous species, should be evaluated for biotechnological applications and economic value of algal resources [16,17,18].

Both FA concentration and profile differ among the variation of biotic and abiotic parameters, as well as with genetic characteristics from each algae [19,20,21]. Thus, the life cycle of each seaweed can also influence the FA content and characterization of algae. This is relevant, particularly, in red seaweeds which present a triphasic life cycle, and each phase has taxonomic characteristics that enables their distinctiveness [22,23,24]. For instance, the red seaweeds *Grateloupia turuturu* and *Chondracanthus teedei* var. *lusitanicus* synthesize different sulfated polysaccharides, according to their life cycle phase [25,26,27,28]. However, to the best of our knowledge, there is no published literature reporting if that also happens regarding their lipid (in particular, fatty acid) content. Still, to explore industrially these marine resources, it is important to understand if their FA composition also varies during the life cycle phase.

On average, seaweeds have a lipid yield between 0.61% and 4.15% dry weight (DW). However, some seaweed species can present higher values, being considered a good source of unsaturated fatty acids [29].

Undeniably, micro and macroalgae are fundamental organisms for the introduction of long-chain PUFA in food webs, as they possess cellular mechanisms to undergo FA elongation, with the production of molecules containing from 14 to 24 carbons [30,31], an ability that most consumers, for example, do not present.

These PUFAs can be beneficial for human health if the ratio ω-6/ω-3 is taken into account in functional foods and nutraceuticals [31]. The impact of these fatty acids on human gene expression is well understood and the ω-6/ω-3 fatty acid ratio is a critical metric for assessing the benefits of PUFAs. A previous study showed that a 3/1 to 5/1 ω-6/ω-3 fatty acid ratio lowers the risk of breast, prostate, colon, and renal cancers [32,33]. In other cases, the ω-6/ω-3 fatty acid ratio of 2/1 to 3/1 was found to minimize inflammation in rheumatoid arthritis patients. A 5/1 ratio, for example, was shown to be effective in asthma patients [32]. In contrast, a fatty acid ratio of 10/1 and higher has been linked to negative outcomes [32,33].

This study aims to evaluate and perform a comparative analysis of the potential of eight algae species harvested from the Central region of Portugal to determine the most suitable seaweeds as a source of FA that can be used as food or a dietary supplement.

## 2. Materials and Methods

### 2.1. Seaweed Harvesting

Throughout the year of 2020, five red seaweeds (*Asparagospis armata*, *Calliblepharis jubata*, *Chondracanthus teedei* var. *lusitanicus Gracilaria gracilis*, and *Grateloupia turuturu*) and three brown seaweeds (*Colpomenia peregrina*, *Sargassum muticum* and *Undaria pinnatifida*) were harvested in two Portuguese seashores (Buarcos Bay, Figueira da Foz, and Quebrado Beach, Peniche) (Table 1). Following that, seaweeds were transported to the laboratory in plastic bags in a coolbox and frozen at −20 °C until further analysis.

After that, the seaweed biomass was washed with filtered seawater to remove sand, epiphytes, and other detritus. Due to the biochemical profile variation according to the life cycle, the red seaweed *G*. *turuturu* and *C*. *teedei* var. *lusitanicus* were differentiated according to their generation through a binocular magnifying glass.

The biomass was then washed with distilled water to eliminate the salt content of seawater, placed in plastic trays, and dried for 48 h at 60 °C in an air-forced oven (Raypa DAF-135, R. Espinar S.L., Barcelona, Spain). Following this, the biological samples were milled (<1 cm) with a commercial grinder (Taurus aromatic, Oliana, Spain) and stored in Eppendorfs in a dark and dry place at room temperature.

### 2.2. Fatty Acid Analysis

Fatty acids were extracted from dry algal biomass and transmethylated to fatty acid methyl esters (FAMEs) for analysis as described by Gonçalves et al. (2012) [34] and stored in liquid form at −80 °C until analysis.

FAMEs identification was performed by Gas chromatography-Mass spectrometry (GC-MS), with resort to a Thermo Scientific Trace 1310 Network (Waltham, MA, USA) equipment, equipped with TR-FFAP column of 0.32 mm internal diameter, 0.25 µm film thickness, and 30 m long. The sample (0.60 µL) was injected at splitless mode, at an injector temperature of 250 °C, lined with a split glass liner of 4.0 mm i.d. The initial oven temperature was 80 °C, following a linear temperature increase of 25 °C min^−1^ to 160 °C, followed by another ramp of 2 °C min^−1^ to 210 °C and finally an increase of 40 °C min^−1^ until a final temperature of 230 °C was reached and maintained for 10 min. Helium at a flow rate of 1.4 mL min^−1^ was used as carrier gas. A Thermo Scientific ISQ 7000 Network Mass Selective Detector at scanning *m*/*z* ranges specific for fatty acids in Selected Ion Monitoring (SIM) mode acquisition was used. The detector starts operating 3.5 min after injection, corresponding to solvent delay. The injector ion source and transfer line were maintained at 240 °C and 230 °C, respectively. Integration of FAME peaks were carried out using the equipment’s software. Identification of each peak was performed by retention time and mass spectrum of each FAME, comparing to the Supelco^®^37 component FAME mix (Sigma-Aldrich, Steinheim, Germany). Quantification of FAMEs was performed as described in Gonçalves et al. (2012) [34].

### 2.3. Statistical Analysis

The fatty acid profiles of the seaweed species studied were statistically analyzed and compared through non-metric multidimensional scaling (n-MDS), associated to analysis of similarities (ANOSIM) and similarity percentage analysis (SIMPER), to assess the similarities and the average dissimilarity between groups, including the contribution (percentage) of each fatty acid to dissimilarities between groups, as well as analysis of variance (ANOVA), to assess differences in the studied components between species.

## 3. Results

The calculated moisture percentage of each algae species is presented in Table 2.

Fatty acid analysis allowed the identification of SFA, MUFA, PUFA, and HUFA in the studied species, with a particular interest in the omega-3 fatty acids encountered, as represented in Table 3 and Table 4. In terms of total fatty acids per gram of dried algae, *Sargassum muticum* was the species presenting the highest value, with the contribution of HUFA for the total FA content being particularly high (of 28% of total FA). Saturated fatty acids were the most abundant class of FA in most species, except for *Undaria pinnatifida* and *Calliblepharis jubata*, where HUFA was the most abundant class. All algae species presented a considerably low ω-6/ω-3 ratio, with the highest of 0.15 in *S. muticum* and the lowest of 0.01 in *C. jubata*.

**Figure 1:**
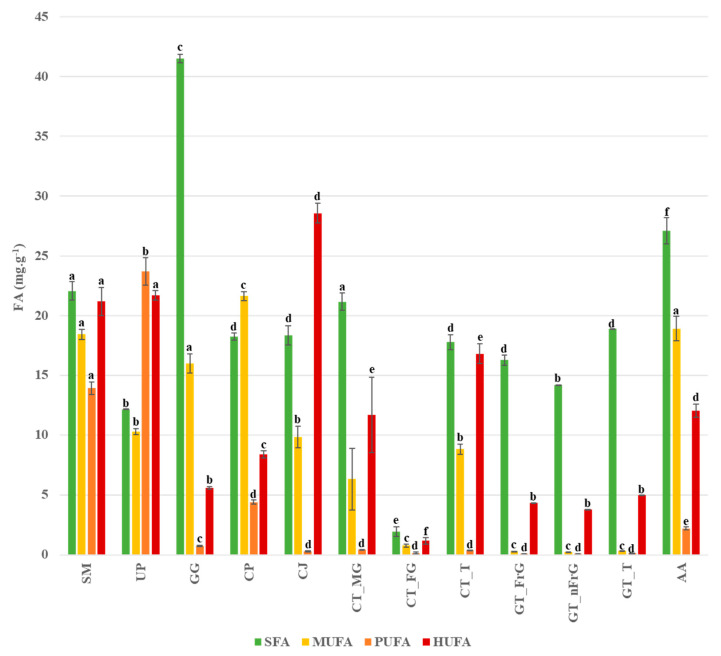
Concentration of the four fatty acids groups (SFA, MUFA, PUFA, HUFA) present in the specimens of the species studied: SM—*Sargassum muticum*, UP—*Undaria pinnatifida*, GG—*Gracilaria gracilis*, CP—*Colpomenia peregrina*, CJ—*Calliblepharis jubata*, CT_MG—*Chondracanthus teedei* var. *lusitanicus* (male gametophyte), CT_GF—*Chondracanthus teedei* var. *lusitanicus* (female gametophyte), CT_T—*Chondracanthus teedei* var. *lusitanicus* (tetrasporophyte), GT_FG—*Grateloupia turuturu* (fructified gametophyte), GT_nFG—*Grateloupia turuturu* (non-fructified gametophyte), GT_T—*Grateloupia turuturu* (tetrasporophyte), AA—*Asparagopsis armata.* Statistically significant differences in the same fatty acid content among the species are expressed by letters above the bars.

**Figure 2:**
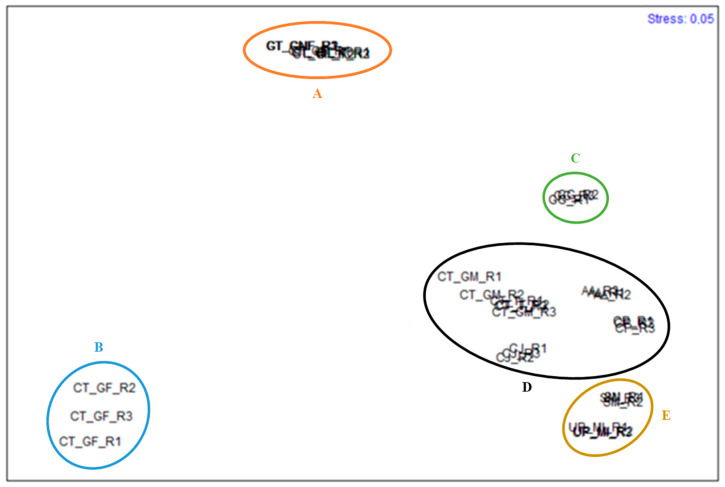
n-MDS of the species studied, regarding the fatty acid profile and content: *Sargassum muticum* (SM_R1, SM_R2, SM_R3), *Undaria pinnatifida* (UP_MI_R1, UP_MI_R2, UP_MI_R3), *Gracilaria gracilis* (GG_R1, GG_R2, GG_R3), *Colpomenia peregrina* (CP_R1, CP_R2, CP_R3), *Calliblepharis jubata* (CJ_R1, CJ_R2, CJ_R3), *Chondracanthus teedei* (male gametophyte) (CT_MG_R1, CT_MG_R2, CT_MG_R3), *Chondracanthus teedei* (female gametophyte) (CT_GF_R1, CT_GF_R2, CT_GF_R3), *Chondracanthus teedei* (tetrasporophyte) (CT_T_R1, CT_T_R2, CT_T_R3), *Grateloupia turuturu* (fructified gametophyte) (GT_FrG_R1, GT_FrG_R2, GT_FrG_R3), *Grateloupia turuturu* (non-fructified gametophyte) (GT_nFrG_R1, GT_nFrG_R2, GT_nFrG_R3), *Grateloupia turuturu* (tetrasporophyte) (GT_T_R1, GT_T_R2, GT_T_R3), *Asparagopsis armata* (AA_R1, AA_R2, AA_R3). Five groups have been identified composed by the following samples: A - GT_FrG_R1, GT_FrG_R2, GT_FrG_R3, GT_nFrG_R1, GT_nFrG_R2, GT_nFrG_R3, GT_T_R1, GT_T_R2, GT_T_R3); B - CT_GF_R1, CT_GF_R2, CT_GF_R3; C—GG_R1, GG_R2, GG_R3; D –CT_MG_R1, CT_MG_R2, CT_MG_R3, CT_T_R1, CT_T_R2, CT_T_R3, CJ_R1, CJ_R2, CJ_R3, CP_R1, CP_R2, CP_R3, AA_R1, AA_R2, AA_R3; and E—SM_R1, SM_R2, SM_R3, UP_MI_R1, UP_MI_R2, UP_MI_R3.

Significant differences between the species studied regarding the FA content of the four FA classes analyzed can be observed in Figure 1. Species are considerably different from each other concerning different fatty acid classes. *Gracilaria gracilis* stands out due to its high content in SFA, while *C. jubata* stands out due to its higher content in HUFA compared to the remaining FA classes; the three forms of *G. turuturu* present an interesting profile, with both SFA and HUFA standing out. Apart from *U. pinnatifida*, PUFA was the FA class in lower concentration compared to the remaining studied species. The n-MDS conducted for the algae species studied is presented in Figure 2.

The results show five different groups: group A, composed of *G. turuturu* replicates, including the stages of fructified gametophyte, non-fructified gametophyte, and tetrasporophyte; group B, including *C. teedei* in the form of female gametophyte replicates; group C, comprising *G. gracilis* replicates; group D, which includes *C. teedei* male gametophyte and tetrasporophyte, *C. jubata* replicates, *C. peregrina* and *A. armata;* and group E, composed of *S. muticum* and *U. pinnatifida* specimens. Significant differences were found between every group. The fatty acids C16:0, α and γ-LA, EPA, and DHA were the most determinant for differentiating the groups. Palmitic acid contributed the most to the dissimilarity between groups D and B (23.20%), C and B (35.68%), B and A (30.90%), and C and A, although in this case, C16:0, C18:1, and C15:1 were equivalently important to differentiate the groups (contributing, respectively, to 24.91%, 24.12%, and 22.35% of the dissimilarity between the groups). DHA, γ-LA and α-LA were, in this order, determining to differentiate groups E and C (contributing to 17.73%, 16.50%, and 16.50%, respectively) and groups E and A (contributing to 14.43%, 13.58%, and 12.64%, respectively). Regarding groups D and A, DHA contributed to 25.31% of the dissimilarity between groups, while contributing to 33.75% to the dissimilarities between groups D and C, as *G. gracilis* does not possess DHA in its profile, while the species of group D do. In groups D and E, γ-LA and α-LA contributed similarly to the similarity, contributing together to 37.05% of the dissimilarity. Groups E and B were differentiated by the contents of EPA, C16:0 and γ-LA and α-LA (contributing to 14.84%, 13.12%, 12.96%, and 12.80%, respectively). The average dissimilarities between groups, as well as the main three FA contributing for the dissimilarities between groups, are presented in Table 5.

Considering each species individually, it is worth noticing the contribution of C16:0 to the total FA content of *G. gracilis*, *C. teedei* var. *lusitanicus* (male gametophyte), and *A. armata*, corresponds to over 60%, 50%, and 43% of the algae’s total FA content, respectively. It is also interesting to note the content of DHA of *C. jubata*, corresponding to circa 50% of the algae’s total FA content. Overall, C16:0 was the most abundant SFA of the studied species, EPA was present in all specimens analyzed, and DHA was the most abundant HUFA, whenever present.

## 4. Discussion

Red and brown seaweeds are particularly well-known sources of ω-3 PUFA and HUFA [35]. Nevertheless, the lipidic profile is specific and a characteristic signature of each seaweed, being dependent on the factors the organism is subjected to [36,37,38], and thus potentially indicative of the conditions that algae has been subjected to. In this context, the geolocation and the exposure to different abiotic (i.e., temperature, salinity, pH, wave exposure, light, nutrient availability) and biotic factors (i.e., herbivory) can lead to different biochemical profiles in the same species, being changes in the FA profile that are particularly noticeable and interesting from a human nutritional perspective [36,39,40,41].

**Figure 3:**
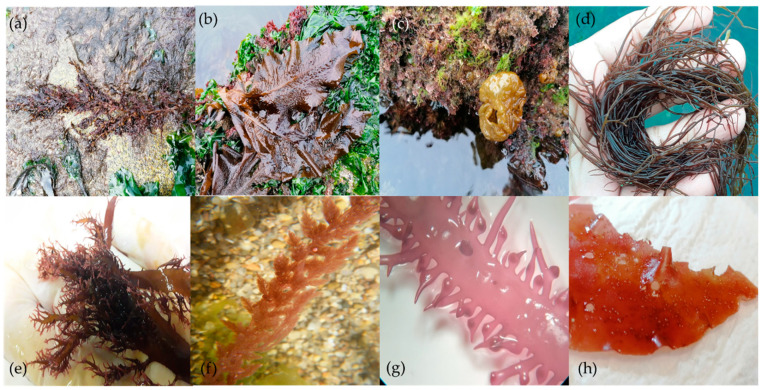
Brown seaweeds (**a**) *Sargassum muticum*, (**b**) *Undaria pinnatifida*, and (**c**) *Colpomenia peregrina*; red seaweeds (**d**) *Gracilaria gracilis*, (**e**) *Calliblepharis jubata*, (**f**) *Asparagopsis armata*, (**g**) *Chondracanthus teedei* var. *lusitanicus* (female gametophyte), and (**h**) *Grateloupia turuturu* (fructified gametophyte).

The brown seaweed *S. muticum* (Figure 3a) is native to Japan and considered an invasive species in Atlantic waters [42,43,44]. Despite the nutritional potential of this seaweed having already been highlighted by several authors [17,19,45], currently, there is no economical exploitation of this species by the food industry [46]. Even though the lipid fraction represents a low part of seaweeds’ constitution, they contain essential fatty acids pivotal for human health [18]. Similarly to the studies conducted by Santos et al. (2020) and Debbarma et al. (2016) [47,48], which revealed that palmitic acid (C16:0) was the most abundant FA present in *S. muticum*, representing, respectively, 24.18% and 43.10% of the algae’s total fatty acid fraction, the present study too presents palmitic acid as the most abundant FA. However, biomass collected in Aguda beach (Porto), during the spring showed a more diverse lipid profile, exhibiting PUFA (such as, C16:2, 20:2 and 20:3), HUFA (like 18:4 ω-3), MUFA (likewise, C20:1 and C22:1), and SFA (namely, C14:0, C15:0, C20:0 and C22:0) [47] that were not detected in our study with *S. muticum* harvested in Buarcos Bay (Figueira da Foz) during the autumn. Previous research showed that in fact, *S. muticum* FA content varies throughout seasons, reaching its maximum yield during the spring and its minimum in the winter [19]. Moreover, it is noted that *S. muticum* content of PUFA reported by Santos et al. (2020) is significantly higher than in this study [47]. A water temperature gradient is observed throughout the Portuguese coast, being registered higher water temperatures in the South of the country and lower in the North. Thus, the FA profile of a same algae species also differs according to the sea water temperature, depending on the zone it is harvested from. For instance, it has been reported that seaweeds collected in zones with colder waters have a higher PUFA content than those of the same species from warmer waters [19], further supporting this discrepancy between a same species harvested from different sampling sites.

For the food industry, the brown seaweed *U. pinnatifida* or wakame (Figure 3b) is one of the most representative sea vegetables, particularly in Asiatic countries (its native area), where their aquaculture production reaches its greatest expression, contributing significantly to seaweeds’ global production and trade [49]. This species holds a high economic value due to its rich nutritional value and human health promoting properties, which stirred the attention of the worldwide food market [50]. However, the cultivation of this species in European waters is not legally allowed, due to their non-indigenous character and invasive behavior [51]. As a result, this invasive species cannot be grown in non-native ecosystems; still, it can be harvested from coastal areas and directly or indirectly introduced into the daily human diet. Due to the widespread presence of this brown seaweed along the European shoreline, it represents a valuable feedstock for the food industry [52]. Furthermore, *U. pinnatifida* contains essential FA for human health promotion, such as EPA, ARA, and DHA [53]. Nevertheless, there are several parameters that can affect this seaweed FA composition and content. For instance, *U. pinnatifida* harvested from the Brittany coast (France) exhibited a much lower amount of total FA (16.6 mg·g^−1^) and an overall different lipidic profile compared to the specimens analyzed in the present study [54]. Moreover, in the southwest coast of Golfo Nuevo (Argentina), *U. pinnatifida* exhibited not only lower values of total FA, SFA, MUFA, and PUFA (18.1, 2, 1, and 15 mg·g^−1^, respectively), but also a different fatty acid profile. For instance, this species presented SFA, such as C14:0, C15:0, C20:0, and C22:0; PUFA, namely C20:3; and HUFA, such as C18:4 and C22:5 (ω-3) [53]. Several studies found that palmitic acid (C16:0) was the most abundant *U. pinnatifida* FA [53,55]; however, in the present study it was found that eicosapentaenoic acid (13.15 mg·g ^−1^) was the most representative, which could be due to different environmental conditions that this algae may be exposed to. Despite all the aforementioned factors that affect the FA concentration and constitution, its profile can also differ according to the part of the seaweed analyzed. For instance, researchers found that the FA profile varies if the analysis is performed on the sporophyll, the frond, or in the midrib of *U. pinnatifida* [53].

The brown seaweed *C. peregrina* (Figure 3c) is a cosmopolitan species that is considered non-indigenous in the Atlantic Ocean [56,57,58]. Despite its nutritional value, this species is still an unexploited resource for the food industry [15,59]. Yet, this brown seaweed’s FA profile and relevance for human nutrition has already been studied. For instance, *C. peregrina* harvested in the Atlantic Ocean (England) showed a diverse lipidic profile, exhibiting the same amount (16.7 mg·g^−1^) of the SFAs C14:0, C16:0, C18:0, and C18:1, and the HUFA C20:4 and C20:5 [59], while in our study, *C. peregrina* not only showed a different FA profile, but also presented different concentrations of each FA.

The species *G. gracilis* (Rhodophyta) (Figure 3d) is among the seaweeds with increasing demand and therefore economic relevance [49]. As a result, increasing research has been conducted to enhance cultivation and evaluate the nutraceutical relevance of this seaweed [60,61,62]. Previous research showed that *G. gracilis* biochemical composition is dependent on the depth it grows. For instance, the total lipid content was higher in the seaweed cultured at a depth of 2.5 m than the seaweed cultivated at 0.5 m [63]. Nevertheless, researchers studied *G. gracilis* FA profile variation during the harvesting season and found that the total content and the lipid (and fatty acid in particular) composition of this seaweed collected at the Lesina lagoon (Italy) is higher and more diverse in the spring and lower and less diverse in the autumn [60]. Despite some differences in the FA profile, our results are in line with Capillo et al. (2018) [64], where the most abundant FA registered were palmitic acid (C16:0) and oleic acid (C18:1). In contrast, these researchers found a high concentration of arachidonic acid (C20:4 ω-6) [64], whereas the mentioned FA was not found in the study presented here.

The native red seaweed *C. jubata* (Figure 3e) is a carrageenan producer, being this molecule pivotal for the food industry, with widespread applications. Yet, *C. jubata* is an unexploited resource as a food product itself or its molecules (i.e., carrageenan) [65,66]. Still, researchers highlight its nutritional potential, particularly as an essential FA source, being the most representative of the palmitic acid (C16:0) and the eicosapentaenoic acid (C20:5 ω-3) from *C. jubata* harvested in France [67]. Concurrently, in our study, *C. jubata* revealed a higher concentration of C22:6 (DHA). Thus, researchers showed that the FA profile of the red seaweed *C. jubata* varies between geographical locations, but also among wild species, laboratory, and inshore cultivated species. For instance, the inshore cultivated *C. jubata* presented an increasingly diverse fatty acid profile, exhibiting a high concentration of total PUFA, in comparison with wild specimens [66]. The results of the total FA, SFA, MUFA, PUFA, and HUFA of *C. jubata* wild specimens collected in the spring at Buarcos Bay (Figueira da Foz) are in line with our results, exhibiting a similar FA composition, but highlighting the presence of the SFA tetradecanoic acid (C14:0) [66], which was not identified in the specimens studied.

In 1920, the red seaweed *A. armata* (Figure 3f), which is native to Australia, was deliberately introduced into Europe due to the high food demand [68,69,70]. In the past, this species has been incorporated in the human daily diet due to its valuable nutritional composition [71]. Among several micronutrients and trace elements that are essential for the proper functioning of the human organism, this non-native seaweed presents nutraceutical potential as a food supplement [71,72]. For instance, the red seaweed *A. armata* harvested in the Algarve coast (South of Portugal) during the spring showed an overall similar sum of SFA, MUFA, and PUFA, presenting a higher concentration of SFA and a lower PUFA amount. Nevertheless, the FA composition showed some differences, namely through the presence of the SFAs C12:0, C14:0, C15:0, and C18:0 [36].

The red seaweed *C. teedei* var. *lusitanicus* (Figure 3g) is an edible seaweed with nutraceutical potential [24,73,74,75]; however, to the best of our knowledge, there is no previous literature reporting this species’ FA characterization, the present study being the first to present such findings.

Despite reports that *G. turuturu* (Figure 3h) is consumed directly in Asian countries, the full nutraceutical potential of this non-native seaweed has yet to be discovered in Europe [76]. Some scientists, on the other hand, are curious about their chemical structure and bioactivities [72,77,78,79,80]. A study conducted with *G. turuturu* harvested in the Nord-East Atlantic coast of France revealed that the FA composition and concentration of each FA varies according to the season in which the seaweed is collected [78,79,81]. In comparison to our study, despite the FA profile differences, the most abundant MUFA and HUFA synthesized in the winter was also C16:0 and C20:5 ω-3, respectively [78,79,81]. Moreover, the storage methods can also influence *G. turuturu* compounds’ concentration and characterization [80].

Compared to terrestrial plants, seaweeds present a wider variety of metabolites with important biological properties, as well as higher abundances of highly unsaturated fatty acids, namely the ω-3 EPA and DHA and the ω-6 ARA, being particularly important for the introduction of such macronutrients in food webs [82]. Thus, seaweeds present a high potential not only as a direct food product, but also for technological applications that can use their biological compounds to produce functional foods [19,82].

Lipids are a varied group with structural, functional, storage, signaling, and transcription factor activities and characteristics, needed for numerous metabolic processes. Omega-3 fatty acids, of which marine algae are important sources, are essential for animal nutrition, as most animals, including humans, are not able to produce them, or, at least, not at the needed rate to meet the metabolic demands. PUFA and HUFA have particular important functions in human metabolism, being crucial for early human developmental stages and contributing to the prevention of cardiovascular diseases as well as obesity and linked morbidities by enhancing lipid and glucose metabolism, and have a protective effect against cancers and inflammatory processes. It is, therefore, important to consume an appropriate amount of such fatty acids, and maintain a balanced diet when it comes to fatty acid intake [60,83,84]. For years, the main source of dietary PUFA and HUFA, especially omega-3 fatty acids, has been marine fish-derived products, but the advances in knowledge concerning the beneficial effects of these compounds to human health have increased the demand for such products, leading to the search for alternative sources, including seaweeds [85,86,87]. Optimum values for omega-3 fatty acids’ daily consumption have been discussed by many health organizations worldwide which, although differing somewhat in recommended quantities, overall propose a combined consumption of EPA and DHA of 250–500 mg per day for healthy adults [88,89]. When analyzing the results obtained in the present study, we confirm that the contribution of each algae to the daily recommended intake values varied depending on the species. The intake of 250 mg of EPA+DHA may be met by ingesting only around 68 g of fresh *Calliblepharis jubata*, or 90 g of *Undaria pinnatifida*, for example, while the same omega-3 intake regarding *Grateloupia turuturu* would take ingesting between 415 to 550 g of fresh algae, depending on its life stage.

The importance of the ω-6/ω-3 FA ratio in human diets has gained relevance over the past few years, given the association of an imbalance of this ratio with the appearance of numerous diseases of cardiovascular, inflammatory, autoimmune, or carcinogenic natures [84,90,91], as well as obesity and associated morbidities [90]. A ω-6/ω-3 fatty acid ratio of 1 is deemed as optimal for human diets, as to prevent the appearance of diseases, but imbalances of this ratio are quite common in diets across the world. The World Health Organization (WHO) recommends a ω-6/ω-3 ratio lower than 10 in diets to prevent deleterious health effects [92]. The most imbalanced diets reported are among the populations of Europe and the United States of America, marked by a high dietary intake of ω-6 fatty acids from a diet rich in vegetable oils, and have been reported to go up 20–50, being less elevated in countries as Japan, where the ratio lies around 12 [33,59]. Nonetheless, these high ratios across the world have some exceptions, as is the case of Greenland Eskimos, whose diets present a ratio of ~1, due to the high consumption of fish that constitute a valuable source of ω-3 fatty acids [85] and contribute to balancing the ratio [93,94]. The fatty acid content of seaweeds is somewhat variable depending on the factors the organisms are subjected to, with the differences particularly noticeable among seasons, as discussed above. In general, algae have been reported to have a ω-6/ω-3, ratio always below the ratio recommended by the WHO, which is 10, attaining minimum ratio values in springtime. The studied algae are concordant with these reports: for instance, *Sargassum* species have been reported elsewhere presenting ratios between 0.55 [95] and 3.37 [19], the latter referring to *S. muticum*, with minimum values during spring months. The species *S. muticum* studied in the present work showed lower values (of 0.15), representing an even better value, probably due to the environmental factors the algae are subjected to in the Portuguese coast, considering that this is a non-indigenous species. Red seaweeds are also reported to rarely surpass the ratio limit of 10—this was reported in Francavilla et al. (2013) [60] referring to *Gracilaria*, where a ratio above 10 was found in specimens collected in January that presented very high amounts of ARA, but the ratio dropped to below this limit in spring, when the maximum content of FA was also observed.

All algae species addressed in the present work present a ω-6/ω-3 ratio far below 1, meaning that their introduction in diets may be beneficial from a nutraceutical perspective, as they provide essential fatty acids needed for numerous metabolic processes while contributing to the lowering of the ω-6/ω-3 ratio of the consumers’ diet, contributing to the prevention of the diseases already mentioned.

In terms of the most suitable algae amongst those studied that would be suitable for food industry applications, it was interesting to note the cumulative potential benefits that would come from the overall low ω-6/ω-3 ratios in combination with low SFA contents (higher PUFA+HUFA/SFA ratios). Although it is known that an exceedingly high PUFA/SFA ratio in diets may be also deleterious to human health, as PUFA are more susceptible to oxidative stress and peroxidation, which may contribute to ageing of tissues and associated morbidities [94,95,96], SFA are present in most foodstuffs of Western diets, and a higher PUFA/SFA ratio in the algae studied could also contribute to the overall FA balance of diets. Thus, we consider that *C. jubata* and *U. pinnatifida* could be the species among the studied that gather the mentioned characteristics, followed by *S. muticum* and *C. teedei* tetrasporophyte, the latter two presenting higher contents of SFA but still attaining high contents of PUFA+HUFA. *G. gracilis, C. teedei* var. *lusitanicus* (female gametophyte), and *G. turuturu* of all three stages seem to be the ones that would contribute the least to the mentioned objectives, by their FA profiles. It would also be interesting to further explore the nutritional and nutraceutical potential of *A. armata*, as it possesses an interesting FA profile, with a rather high content in the omega-3 fatty acids EPA and DHA. In addition, other studies have reported that some of the algae addressed in this study possess other high-quality components, such as polysaccharides, micronutrients, trace elements and vitamins [97,98]. It would, then, be interesting to undergo future studies with the studied algae to determine the potential effects that their other components may have on human health, both from positive and negative perspectives, to provide more complete information and the range of possibilities concerning the overall potential of algae as alternative sources that may be exploited by the food and nutraceutical industries.

## 5. Conclusions

Both red and brown seaweeds are a reservoir of bioactive compounds with several biotechnological applications. Among algal compounds, PUFA are essential components of human nutrition and are considered to have a variety of health benefits. Dietary PUFA consumption, which contains both ω-3 and ω-6 FA, has been shown to affect inflammatory processes and other cell functions, with the understanding of the ω-6/ω-3 ratio proven to be essential to predict possible health implications of the consumption of the algae, or of foods in general.

*Calliblepharis jubata* and *Undaria pinnatifida* were the algae among those harvested proving to be the most suitable for food industry applicability in terms of the potential nutraceutical benefits they present. Both algae present high contents in polyunsaturated and highly unsaturated fatty acids, with ω-3 FA being the most prominent, as may also be confirmed given their low ω-6/ω-3 ratios (of 0.01 and 0.09, respectively). Although all studied algae present very low ω-6/ω-3 ratios, which may contribute to the prevention of diseases, *C. jubata* and *U. pinnatifida* combine that characteristic with SFA contents lower that PUFA+HUFA contents, which does not happen in the remaining species.

Regardless of the nutraceutical potential of seaweeds, it is crucial to ensure their long-term development through cultivation techniques that ensure the final product’s safety and quality. Furthermore, seaweed processing and transformation must be considered in order to maintain the FA profile and concentration, as well as the stability of these important components. From an industrial and commercial standpoint, it is essential to ensure that the nutraceutical potential is given from the cultivation/harvesting to the final product.

## Figures and Tables

**Table 1:** Seaweed harvesting sites and dates.

Seaweed Species	Location	GPS Location	Harvesting Date
Rhodophyta (red seaweed)			
Asparagopsis armata *	Quebrado Beach	39.368258, −9.372303	20/10/2020
*Calliblepharis jubata*	Buarcos Bay	40.165867, −8.885556	19/10/2020
*Chondracanthus teedei* var. *lusitanicus*	Buarcos Bay	40.165867, −8.885556	27/05/2020
*Gracilaria gracilis*	Buarcos Bay	40.165867, −8.885556	19/10/2020
Grateloupia turuturu *	Buarcos Bay	40.165867, −8.885556	13/01/2020
Ochrophyta (brown seaweed)			
Colpomenia peregrine *	Quebrado Beach	39.368258, −9.372303	20/10/2020
Sargassum muticum *	Buarcos Bay	40.165867, −8.885556	19/10/2020
Undaria pinnatifida *	Buarcos Bay	40.165867, −8.885556	13/01/2020

* non-indigenous seaweed species.

**Table 2:** Moisture (expressed in percentage) of *Sargassum muticum*, *Undaria pinnatifida*, *Colpomenia peregrina*, *Gracilaria gracilis*, *Calliblepharis jubata* and *Asparagopsis armata*, *Chondracanthus teedei* var. *lusitanicus* (MG—male gametophyte, FG—female gametophyte, and T—tetrasporophyte), and *Grateloupia turuturu* (FrG—fructified gametophyte, nFrG—non-fructified gametophyte, and T—tetrasporophyte).

Species	Moisture (%)
*S. muticum*	87.22
*U. pinnatifida*	86.84
*C. peregrina*	91.25
*G. gracilis*	49.49
*C. jubata*	89.13
*A. armata*	87.22
*C. teedei* (MG)	85.86
*C. teedei* (FG)	85.43
*C. teedei* (T)	84.35
*G. turuturu* (FrG)	86.78
*G. turuturu* (nFrG)	87.69
*G. turuturu* (T)	88.24

**Table 3:** Fatty acid profile of each fatty acid (expressed in mg·g^−1^ of dried algae) of *Sargassum muticum*, *Undaria pinnatifida, Colpomenia peregrina, Gracilaria gracilis, Calliblepharis jubata*, and *Asparagopsis armata*. α-LA alpha linoleic acid; γ-LA—gamma linoleic acid; ARA—arachidonic acid; EPA—eicosapentaenoic acid; DHA—docosahexaenoic acid. Results are expressed in mean ± standard deviation. The sum of fatty acids’ (FA) content that compose each class (SFA—saturated fatty acids; MUFA—monounsaturated fatty acids; PUFA—polyunsaturated fatty acids; HUFA—highly unsaturated fatty acids), the ratio of omega 6/omega 3 and the *N*—diversity in FA molecules have been highlighted with bold format in the respective table lines.

	*S. muticum*	*U. pinnatifida*	*C. peregrina*	*G. gracilis*	*C. jubata*	*A. armata*
C16:0	20.89 ± 0.72	11.51 ± 0.01	18.24 ± 0.31	40.46 ± 0.23	15.94 ± 0.73	26.39 ± 1.19
C17:0	0.29 ± 0.03	0.00 ± 0.00	0.00 ± 0.00	0.00 ± 0.00	2.24 ± 0.05	0.00 ± 0.00
C18:0	0.43 ± 0.03	0.64 ± 0.02	0.00 ± 0.00	1.03 ± 0.13	0.17 ± 0.03	0.71 ± 0.10
C24:0	0.45 ± 0.05	0.00 ± 0.00	0.00 ± 0.00	0.00 ± 0.00	0.00 ± 0.00	0.00 ± 0.00
**∑ SFA**	**22.06**	**12.15**	**18.24**	**41.49**	**18.35**	**27.10**
C15:1	3.15 ± 0.15	2.44 ± 0.10	7.72 ± 0.14	5.64 ± 0.47	3.28 ± 0.14	8.53 ± 0.29
C16:1	7.43 ± 0.15	1.66 ± 0.06	2.66 ± 0.05	1.13 ± 0.09	2.14 ± 0.20	3.60 ± 0.18
C18:1	7.84 ± 0.24	6.21 ± 0.11	11.24 ± 0.28	9.21 ± 0.23	4.42 ± 0.63	6.77 ± 0.56
**∑ MUFA**	**18.42**	**10.31**	**21.62**	**15.98**	**9.83**	**18.90**
C18:2	4.67 ± 0.23	3.87 ± 0.08	0.96 ± 0.06	0.74 ± 0.06	0.27 ± 0.05	0.78 ± 0.04
C18:3 (α-LA)	6.23 ± 0.23	7.51 ± 0.42	1.53 ± 0.01	0.00 ± 0.00	0.00 ± 0.00	0.80 ± 0.09
C18:3 (γ-LA)	3.01 ± 0.24	12.33 ± 0.67	1.92 ± 0.09	0.00 ± 0.00	0.00 ± 0.00	0.62 ± 0.01
**∑ PUFA**	**13.92**	**23.71**	**4.40**	**0.74**	**0.27**	**2.21**
C20:4 (ARA)	0.00 ± 0.00	0.00 ± 0.00	0.00 ± 0.00	0.00 ± 0.00	0.00 ± 0.00	0.77 ± 0.05
C20:5 (EPA)	13.83 ± 0.48	13.15 ± 0.02	2.59 ± 0.00	5.58 ± 0.09	6.22 ± 0.22	2.43 ± 0.01
C22:6 (DHA)	7.33 ± 0.72	8.55 ± 0.37	5.80 ± 0.29	0.00 ± 0.00	22.34 ± 0.63	8.83 ± 0.49
**∑ HUFA**	**21.17**	**21.70**	**8.39**	**5.58**	**28.56**	**12.03**
**∑ FA**	**75.56**	**67.86**	**52.65**	**63.80**	**57.01**	**60.23**
**ω-6/** **ω-3**	**0.15**	**0.09**	**0.08**	**0.13**	**0.01**	**0.12**
***N***	**12**	**10**	**9**	**7**	**9**	**11**

**Table 4:** Fatty acid profile of each fatty acid (expressed in mg·g^−1^ of dried algae) of *Chondracanthus teedei* var. *lusitanicus* (MG—male gametophyte, FG—female gametophyte, and T—tetrasporophyte) and *Grateloupia turuturu* (FrG—fructified gametophyte, nFrG—non-fructified gametophyte, and T—tetrasporophyte). Results are expressed in mean ± standard deviation. The sum of fatty acids’ (FA) content that compose each class (SFA—saturated fatty acids; MUFA—monounsaturated fatty acids; PUFA—polyunsaturated fatty acids; HUFA—highly unsaturated fatty acids), the ratio of omega 6/omega 3 and the *N*—diversity in FA molecules have been highlighted with bold format in the respective table lines.

	*C. teedei* (MG)	*C. teedei* (FG)	*C. teedei* (T)	*G. turuturu* (FrG)	*G. turuturu* (nFrG)	*G. turuturu* (T)
C16:0	20.29 ± 8.45	1.66 ± 4.36	17.46 ± 0.63	13.59 ± 14.20	11.84 ± 5.65	15.75 ± 5.41
C17:0	0.00 ± 0.00	0.00 ± 0.00	0.00 ± 0.00	0.00 ± 0.00	0.00 ± 0.00	0.00 ± 1.08
C18:0	0.50 ± 0.04	0.12 ± 0.07	0.32 ± 0.02	2.69 ± 0.49	2.34 ± 0.00	3.12 ± 0.10
C24:0	0.26 ± 0.20	0.15 ± 0.04	0.00 ± 0.00	0.00 ± 0.00	0.00 ± 0.00	0.00 ± 0.00
**∑ SFA**	**21.16**	**1.93**	**17.78**	**16.28**	**14.18**	**18.87**
C15:1	2.41 ± 0.93	0.21 ± 0.48	2.63 ± 0.34	0.00 ± 0.00	0.00 ± 0.00	0.00 ± 0.00
C16:1	0.84 ± 0.03	0.12 ± 0.10	1.20 ± 0.06	0.05 ± 0.84	0.05 ± 0.88	0.06 ± 0.81
C18:1	3.08 ± 2.71	0.44 ± 1.29	4.99 ± 0.28	0.23 ± 3.02	0.20 ± 3.51	0.26 ± 3.37
**∑ MUFA**	**6.32**	**0.77**	**8.82**	**0.28**	**0.24**	**0.33**
C18:2	0.41 ± 0.01	0.14 ± 0.13	0.38 ± 0.02	0.07 ± 0.27	0.06 ± 0.34	0.08 ± 0.35
C18:3 (α-LA)	0.00 ± 0.00	0.00 ± 0.00	0.00 ± 0.00	0.02 ± 0.64	0.02 ± 0.52	0.03 ± 0.65
C18:3 (γ-LA)	0.00 ± 0.00	0.00 ± 0.00	0.00 ± 0.00	0.00 ± 0.77	0.00 ± 0.72	0.00 ± 0.88
**∑ PUFA**	**0.41**	**0.14**	**0.38**	**0.09**	**0.08**	**0.11**
C20:4 ω-6 (ARA)	0.00 ± 0.00	0.00 ± 0.00	0.00 ± 0.00	0.24 ± 0.00	0.21 ± 0.00	0.28 ± 0.00
C20:5 ω-3 (EPA)	2.66 ± 2.35	0.40 ± 1.11	6.00 ± 0.31	4.08 ± 2.01	3.55 ± 0.89	4.73 ± 2.33
C22:6 ω-3 (DHA)	5.16 ± 4.54	0.76 ± 2.42	10.81 ± 0.55	0.00 ± 2.27	0.00 ± 1.96	0.00 ± 9.07
**∑ HUFA**	**11.70**	**1.16**	**16.81**	**4.32**	**3.76**	**5.01**
**∑ FA**	**39.59**	**4.01**	**43.79**	**30.21**	**26.33**	**35.01**
**ω-** **6/** **ω-** **3**	**0.04**	**0.12**	**0.02**	**0.08**	**0.08**	**0.08**
**N**	**9**	**9**	**8**	**8**	**8**	**8**

**Table 5:** SIMPER results regarding dissimilarities (diss.) between the groups identified (A—*G. turuturu* fructified gametophyte, non-fructified gametophyte, and tetrasporophyte; B—*C. teedei* female gametophyte; C—*G. gracilis*; D—*C. teedei* male gametophyte and tetrasporophyte, *C. jubata*, *C. peregrina*, and *A. armata*; E—*S. muticum* and *U. pinnatifida*), presenting the average dissimilarity (Av. diss.) between groups, the three FA that contribute the most for dissimilarities between groups, including the percentage of contribution to that dissimilarity and the cumulative contribution of those three FA for the total dissimilarity between groups, in percentage.

Groups	Av. diss. between Groups	Main FA	% Contribution to diss.	% Cumulative Contribution to diss.
**D,E**	37.98	EPA	19.96	49.75
		γ-LA	15.85	
		α-LA	13.94	
**D,C**	38.58	C16:0	47.58	81.64
		DHA	24.89	
		C18:1	9.17	
**E,C**	50.01	C16:0	36.05	59.46
		DHA	11.74	
		EPA	11.67	
**D,B**	85.65	C16:0	39.13	72.93
		DHA	21.86	
		C18:1	11.94	
**E,B**	89.62	C16:0	21.14	52.14
		EPA	19.32	
		γ-LA	11.67	
**C,B**	90.88	C16:0	63.01	86.06
		C18:1	14.24	
		C15:1	8.81	
**D,A**	45.84	DHA	29.60	58.05
		C16:0	15.38	
		EPA	13.08	
**E,A**	53.45	EPA	15.48	44.40
		DHA	14.56	
		γ-LA	14.35	
**C,A**	51.04	C16:0	54.82	79.58
		C18:1	14.44	
		EPA	10.33	
**B,A**	82.71	C16:0	45.61	75.62
		EPA	21.40	
		ARA	8.61	

## Data Availability

The data reported in the present study is compared and discussed in relation to other published works on the subject, as identified and cited throughout the manuscript. There was no resort to publicly available datasets.
